# Serum Uric Acid, Alzheimer-Related Brain Changes, and Cognitive Impairment

**DOI:** 10.3389/fnagi.2020.00160

**Published:** 2020-06-05

**Authors:** Jee Wook Kim, Min Soo Byun, Dahyun Yi, Jun Ho Lee, So Yeon Jeon, Kang Ko, Gijung Jung, Han Na Lee, Jun-Young Lee, Chul-Ho Sohn, Yun-Sang Lee, Seong A Shin, Yu Kyeong Kim, Dong Young Lee, for the KBASE Research Group

**Affiliations:** ^1^Department of Neuropsychiatry, Hallym University Dongtan Sacred Heart Hospital, Hwaseong, South Korea; ^2^Department of Psychiatry, Hallym University College of Medicine, Chuncheon, South Korea; ^3^Institute of Human Behavioral Medicine, Medical Research Center Seoul National University, Seoul, South Korea; ^4^Department of Neuropsychiatry, Seoul National University Hospital, Seoul, South Korea; ^5^Department of Neuropsychiatry, Chungnam National University Hospital, Daejeon, South Korea; ^6^Department of Geriatric Psychiatry, National Center for Mental Health, Seoul, South Korea; ^7^Department of Neuropsychiatry, SMG-SNU Boramae Medical Center, Seoul, South Korea; ^8^Department of Psychiatry, Seoul National University College of Medicine, Seoul, South Korea; ^9^Department of Radiology, Seoul National University Hospital, Seoul, South Korea; ^10^Department of Nuclear Medicine, Seoul National University College of Medicine, Seoul, South Korea; ^11^Department of Nuclear Medicine, SMG-SNU Boramae Medical Center, Seoul, South Korea

**Keywords:** serum uric acid, Alzheimer’s disease, cerebral glucose metabolism, cognitive impairment, neurodegeneration

## Abstract

**Background:**

Despite known associations of lower serum uric acid (UA) with Alzheimer’s disease (AD) dementia or AD-related cognitive impairment, little is known regarding the underlying patho-mechanisms. We aimed to examine the relationships of serum UA with in vivo AD pathologies including cerebral beta-amyloid (Aβ) and tau deposition, AD-signature region cerebral glucose metabolism (AD-CM), and white matter hyperintensities (WMH). We also investigated the association between serum UA and cognitive performance, and then assessed whether such an association is mediated by the brain pathologies.

**Methods:**

A total of 430 non-demented older adults underwent comprehensive clinical assessments, measurement of serum UA level, and multimodal brain imaging, including Pittsburgh compound B-positron emission tomography (PET), AV-1451 PET, fluorodeoxyglucose (FDG)-PET, and magnetic resonance imaging scans. Mini-Mental State Examination (MMSE) and word list recall (WLR) test scores were used to measure cognitive performance.

**Results:**

Serum UA level was significantly associated with AD-CM, but not with Aβ deposition, tau deposition, or WMH volume. Serum UA levels also had significant association with WLR and marginal association with MMSE; such associations disappeared when AD-CM was controlled as a covariate, indicating that AD-CM has a mediating effect.

**Conclusion:**

The findings of the present study indicate that there is an association of low serum UA with AD-related cerebral hypometabolism, and whether this represents a causal relationship remains to be determined.

## Introduction

Uric acid (UA) is a naturally produced water-soluble antioxidant, which contributes more than half of the free radical scavenging activity in the peripheral nervous system (Ames et al., 1981; Choi et al., 2005; Gao et al., 2008). UA is also regarded as a potential central nervous system antioxidant (Bowman et al., 2010), and its protective effects have been reported in animal models or in vitro cell models of Parkinson’s disease (Chen et al., 2012) and in a mouse model of multiple sclerosis (Hooper et al., 1998).

An emerging body of epidemiological studies have indicated that a lower serum UA level is associated with poorer cognitive function and increased risk of mild cognitive impairment (MCI) or overall dementia (Euser et al., 2009; Hong et al., 2015; Liu et al., 2017), although a couple of studies did not find such association (Schretlen et al., 2007; Latourte et al., 2018). Regarding its specific relationship with Alzheimer’s disease (AD) dementia, one study reported that serum UA was lower in AD dementia patients than in cognitively normal (CN) healthy controls (Cankurtaran et al., 2013). Moreover, several studies showed that lower serum UA was associated with a markedly higher risk of progressing to AD dementia from a non-demented state (Rinaldi et al., 2003; Euser et al., 2009; Irizarry et al., 2009; Du et al., 2016).

Despite such associations of lower serum UA with AD dementia or AD-related cognitive impairment, little is known regarding the underlying patho-mechanisms. Some in vivo animal cell culture and postmortem human brain cell studies suggested that UA may act as an antioxidant to mitigate Aβ-induced neuronal injury (Keller et al., 1998) or to reduce synaptic dysfunction (Ansari and Scheff, 2010) in AD. However, few studies have investigated *in vivo* neuropathological changes that could link lower serum UA and AD-related cognitive decline.

In this context, we first aimed to investigate the relationships of serum UA with *in vivo* AD pathologies including cerebral Aβ and tau deposition, AD-signature region cerebral glucose metabolism (AD-CM), and white matter hyperintensities (WMH) in non-demented older adults. Secondly, we investigated the association between lower serum UA and cognitive impairment, and then assessed whether such an association is mediated by brain pathologies that had significant relationships with lower serum UA.

## Materials and Methods

### Participants

This study was part of the Korean Brain Aging Study for Early Diagnosis and Prediction of Alzheimer’s Disease (KBASE), which is an ongoing prospective cohort study (Byun et al., 2017). As of February 2017, a total of 430 non-demented (291 CN and 139 MCI) individuals between 55 and 90 years of age were enrolled in the study. The CN group consisted of participants with a Clinical Dementia Rating (CDR) (Morris, 1993) score of 0 and no diagnosis of MCI or dementia. All individuals with MCI met the current consensus criteria for amnestic MCI, which are as follows: (1) memory complaints confirmed by an informant; (2) objective memory impairments; (3) preservation of global cognitive function; (4) independence in functional activities; and (5) no dementia. Regarding Criterion 2, the age-, education-, and sex-adjusted *z*-score was <−1.0 for at least one of four episodic memory tests: Word List Memory, Word List Recall (WLR), Word List Recognition, and Constructional Recall; these are included in the Korean version of the Consortium to Establish a Registry for Alzheimer’s Disease (CERAD-K) neuropsychological battery (Lee et al., 2004). All MCI individuals had a CDR score of 0.5.

The exclusion criteria were as follows: (1) presence of a major psychiatric illness; (2) significant neurological or medical conditions, or comorbidities that could affect mental function; (3) contraindications for a magnetic resonance imaging (MRI) scan (e.g., pacemaker or claustrophobia); (4) illiteracy; (5) the presence of significant visual/hearing difficulties and/or severe communication or behavioral problems that would interfere with clinical examinations or brain scans; (6) use of an investigational drug; and (7) pregnancy or lactation. Further information regarding the recruitment of the KBASE cohort was described in a previous report by our research group (Byun et al., 2017). The study protocol was approved by the Institutional Review Boards of Seoul National University Hospital and SNU-SMG Boramae Center, Seoul, South Korea. All participants provided written informed consent.

### Clinical Assessments

All participants underwent comprehensive clinical and neuropsychological assessments administered by trained psychiatrists and neuropsychologists based on the KBASE assessment protocol (Byun et al., 2017), which incorporates the CERAD-K neuropsychological battery (Morris et al., 1989; Lee et al., 2002). The z-scores of the Mini-Mental State Examination (MMSE) and WLR test, which are included in the CERAD-K neuropsychological battery (Lee et al., 2004), were selected as measurements of global cognitive function and delayed recall ability, respectively. For both tests, lower *z*-score indicates poorer cognitive function (Lee et al., 2004). Importantly, delayed recall impairment is the earliest cognitive change observed in AD (Howieson et al., 1997; Grober et al., 2000).

The comorbidity of vascular risk factors (e.g., hypertension, diabetes mellitus, dyslipidemia, coronary heart disease, transient ischemic attack, and stroke) was assessed based on data collected by trained nurses during systematic interviews of participants and their informants; a vascular risk score (VRS) was calculated based on the number of vascular risk factors present and reported as a percentage (Decarli et al., 2004). Smoking status (never/ex-smoker/smoker) and alcohol intake status (never/former/drinker) were also evaluated by interview.

### Laboratory Tests of Blood Samples

After an overnight fast, blood samples were obtained via venipuncture in the morning (8–9 a.m.). Serum levels of UA were measured using a colorimetry method (ADVIA 1800 Auto Analyzer, Siemens, Washington, DC, United States). The normal ranges for serum UA are 3.7–9.2 g/dL in men and 3.1–7.8 g/dL in women. Serum albumin was also measured using the same method to evaluate a general nutritional state. Additionally, genomic DNA was extracted from whole blood and apolipoprotein E (*APOE*) genotyping was performed as previously described (Wenham et al., 1991). APOE ε4 (*APOE4*) positivity was defined as the presence of at least one ε4 allele.

### Measurement of Cerebral Aβ Deposition

All participants underwent simultaneous three-dimensional (3D) [^11^C] Pittsburgh compound B (PiB)-positron emission tomography (PET) and 3D T1-weighted MRI scan using a 3.0T Biograph mMR (PET-MR) scanner (Siemens), in accordance with the manufacturer’s guidelines. The details of the PiB-PET imaging acquisition and preprocessing were described previously (Park et al., 2019). An automatic anatomical labeling algorithm and a region-combining method (Reiman et al., 2009) were applied to determine regions of interest (ROIs) for characterization of PiB retention levels in the frontal, lateral parietal, posterior cingulate-precuneus, and lateral temporal regions. Standardized uptake value ratio (SUVR) values for each ROI were calculated by dividing the mean value for all voxels within each ROI by the mean cerebellar uptake value in the same image. A global cortical ROI consisting of the four ROIs was defined and a global Aβ retention value was generated by dividing the mean value for all voxels of the global cortical ROI by the mean cerebellar uptake value in the same image (Reiman et al., 2009; Choe et al., 2014). Each participant was classified as Aβ-positive (Aβ+) if the SUVR value was > 1.4 in at least one of the four ROIs or as Aβ-negative (Aβ-) if the SUVR value was ≤ 1.4 for all four ROIs (Reiman et al., 2009; Jack et al., 2014).

### Measurement of Cerebral Tau Deposition

A subset of subjects (*n* = 107) underwent [^18^F] AV-1451 PET scans (Siemens) using a Biograph True point 40 PET/CT scanner (Siemens), in accordance with the manufacturer’s guidelines. While all the other neuroimaging scans were performed during the baseline visit, AV-1451 PET imaging was performed at an average of 2.6 (standard deviation 0.3) years after the baseline visit. The details of AV-1451 PET imaging acquisition and preprocessing were described previously (Park et al., 2019). To estimate cerebral tau deposition, we quantified AV-1541 SUVR of an *a priori* ROI of “AD-signature regions” of tau accumulation, which comprised a size-weighted average of partial volume-corrected uptake in entorhinal, amygdala, parahippocampal, fusiform, inferior temporal, and middle temporal ROIs, in accordance with the method used in a previous report (Jack et al., 2017). The AV-1541 SUVR of the abovementioned ROI was used as an outcome variable for cerebral tau deposition.

### Measurement of AD-Signature Region Cerebral Glucose Metabolism

All participants underwent FDG-PET imaging using the above-described PET-MR machine; the details of FDG-PET image acquisition and preprocessing were described previously (Park et al., 2019). AD-signature FDG ROIs that are sensitive to the changes associated with AD, such as the angular gyri, posterior cingulate cortex, and inferior temporal gyri (Jack et al., 2014), were determined. AD-CM was defined as the voxel-weighted mean SUVR extracted from the AD-signature FDG ROIs; the details of the MRI acquisition and preprocessing were described previously (Park et al., 2019).

### Measurement of WMH

All participants underwent MRI scans with fluid-attenuated inversion recovery (FLAIR) using the abovementioned 3.0T PET-MR scanner in a validated automatic procedure that has previously been reported (Tsai et al., 2014). Briefly, the procedure consists of 11 steps: spatial coregistration of T1 and FLAIR images, fusion of T1 and FLAIR images, segmentation of T1 images, acquisition of transformation parameters, deformation and acquisition of the white matter mask, acquisition of FLAIR within the white matter mask, intensity normalization of the masked FLAIR, nomination of candidate WMH with a designated threshold, creation of a junction map, and elimination of the junction. There were two modifications in the current processing procedure relative to that used in the original study: (a) an optimal threshold of 70 was applied, as it was more suitable for our data than the threshold of 65 that was used in the original study; and, (b) given that individuals with acute cerebral infarcts were not enrolled in our sample, we did not use diffusion-weighted imaging in the current automated procedure. Using the final WMH candidate image, WMH volume was extracted in the native space in each subject.

### Statistical Analysis

To examine relationships between serum UA and neuroimaging biomarkers, multiple logistic regression analyses, linear regression analyses, or general linear model (GLM) analysis with post-hoc tests using the least significant difference (LSD) method were performed as appropriate. Serum UA, as an independent variable for each analysis, was first analyzed as a continuous variable, and then as a stratified categorical variable; subjects were divided into three strata [2.3–4.5 mg/dL (low level), 4.6–5.5 mg/dL (middle level), and 5.6–10.4 mg/dL (high level)] based on the tertiles of the serum UA level. For each analysis of associations between UA and AD neuroimaging biomarkers, three models were tested for stepwise control of potential confounders. The first model did not include any covariates, the second model included age and sex as covariates, and the third model included all potential covariates (i.e., age, sex, education, *APOE4* positivity, VRS, clinical diagnosis [CN vs. MCI], serum albumin, body mass index, smoking status, and alcohol intake status) that might affect the relationship between serum UA and neuroimaging biomarkers (Liberopoulos et al., 2004; Lain et al., 2005; Haj Mouhamed et al., 2011; Towiwat and Li, 2015). For neuroimaging biomarkers that showed significant associations with UA in the above analyses, we performed further multiple linear regression analyses that included a serum UA × age (or sex or *APOE4* or VRS or clinical diagnosis) interaction term, as well as serum UA and age (or sex or *APOE4* or VRS or clinical diagnosis) as independent variables; the neuroimaging biomarker was used as a dependent variable, and the analyses were controlled for age, sex, education, *APOE4*, VRS, and clinical diagnosis as covariates. Additionally, to investigate the association between serum UA and cognitive performance, the z-score differences of the MMSE and WLR were tested among the three UA strata by GLM analysis with post hoc tests using the LSD method. The same GLM analyses were performed with control of AD neuroimaging biomarkers (those that showed significant associations with UA) as covariates, to investigate whether the associations between UA and cognitive function measurements by MMSE or WLR are mediated by those AD biomarkers. All statistical analyses were performed using IBM SPSS Statistics software (version 24, IBM Corp., Armonk, NY, United States). The level of statistical significance was set as a two-tailed *p*-values < 0.05.

## Results

### Participants

Demographic and clinical characteristics of the participants are presented in Table 1; of the total 429 participants, 143 individuals had low serum UA levels, 140 had middle UA serum levels, and 146 had high serum UA levels.

**TABLE 1:** Participant characteristics by serum UA strata (*N* = 429).

Characteristic	High	Middle	Low	χ^2^ or F	*p*
N	146	140	143		
Serum UA	6.56 (0.94)	5.05 (0.27)	3.84 (0.52)	640.426	<0.001
Age, y	70.61 (8.22)	70.30 (8.16)	70.86 (7.68)	0.173	0.841
Female, %	32. 19	61.43	74.83	55.812	<0.001
Education, y	11.90 (4.27)	11.34 (4.95)	10.29 (3.88)	4.297	0.014
WLR					
MMSE	26.06 (3.21)	25.55(3.53)	24.76 (3.59)	5.271	0.005
APOE4 positivity, %	23.97	19.29	26.57	2.156	0.340
Clinical diagnosis, CN, %	71.92	70.00	60.84	4.595	0.101
Body mass index, kg/m^2^	24.97 (2.89)	24.38 (2.89)	22.78 (3.22)	5.705	0.004
Vascular risk score	18.84 (16.41)	15.12 (14.81)	19.23 (17.39)	2.755	0.065
GDS score	6.57 (6.47)	5.87 (6.02)	7.08 (6.22)	1.344	0.262
Serum albumin	4.48 (0.24)	4.48 (0.23)	4.45 (0.24)	1.151	0.317
**Smoking status, %**				33.216	<0.001
Never	50.34	71.43	80.42		
Former	44.14	23.57	15.38		
Smoker	5.52	5.00	4.20		
**Alcohol intake status, %**				30.118	<0.001
Never	37.93	55.00	69.23		
Former	18.62	10.71	11.19		
Drinker	43.45	34.29	19.58		
**AD neuroimage biomarkers**					
**Cerebral A**β **deposition**					
Aβ positivity, %	23.24	26.09	22.70	0.506	0.776
Aβ retention, SUVR	1.28 (0.36)	1.29 (0.35)	1.28 (0.35)	0.069	0.934
**Cerebral tau deposition**^**b**^					
AV-1451, SUVR	1.53 (0.78)	1.59 (0.76)	1.68 (0.83)	0.266	0.767
**Neurodegeneration**^**c**^					
AD-CM, SUVR	1.42 (0.14)	1.39 (0.12)	1.38 (0.12)	3.602	0.028
WMH volume, cm^3^ ^d^	6.31 (6.08)	5.48(5.00)	6.20 (5.06)	0.833	0.436

*Abbreviations: UA, uric acid; WLM, word list recall; MMSE, mini-mental state examination; APOE4, apolipoprotein ε4; CN, cognitively normal; GDS, Geriatric depression scale; Aβ, beta-amyloid; AD, Alzheimer’s disease; AD-CM, Alzheimer’s disease signature cerebral glucose metabolism; SUVR, standardized uptake value ratio; WMH, white matter hyperintensities. ^*a*^Unless otherwise indicated, data are expressed as mean (standard deviation). ^*b*^Number of subjects = 107 (33 in high UA; 44 in middle UA; 30 in low UA), performed after an average of 2.6 (standard deviation 0.3) years from the baseline visit. ^*c*^Number of subjects = 412 (140 in high UA; 135 in middle UA; 137 in low UA). ^*d*^Number of subjects = 377 (132 in high UA; 118 in middle UA; 127 in low UA).*

### Association of Serum UA With Neuroimaging Biomarkers

**FIGURE 1:**
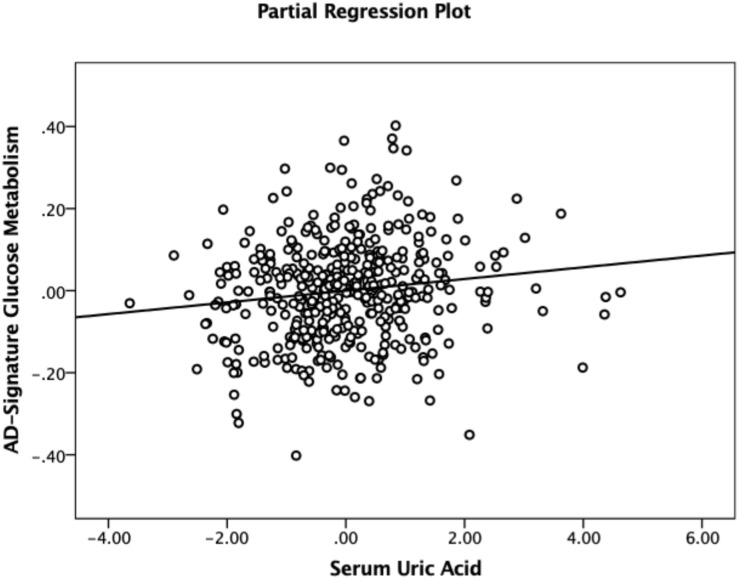
Partial regression plot showing the relationship between serum uric acid and AD-signature cerebral glucose metabolism in non-demented participants. Multiple linear regression analysis was performed after adjusting for age, sex, education, apolipoprotein ε4, vascular risk score, clinical diagnosis, serum albumin, body mass index, smoking status, and alcohol intake status. Abbreviations: AD, Alzheimer’s disease.

**FIGURE 2:**
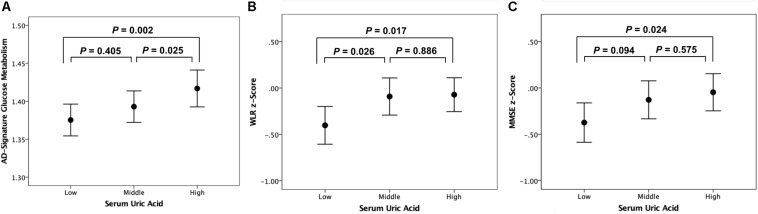
Error bar charts with standard error bars displaying **(A)** AD-signature cerebral glucose metabolism, **(B)** WLR z-scores, and **(C)** MMSE z-scores before adjustment for cerebral glucose metabolism according to serum uric acid strata in non-demented participants. Abbreviations: AD, Alzheimer’s disease; WLR, Word List Recall; MMSE, Mini-Mental State Examination.

As shown in Tables 2, 3, Aβ biomarkers (Aβ positivity and Aβ deposition) and tau deposition both showed no association with serum UA levels. In addition, serum UA was not associated with WMH. In contrast, serum UA concentration showed a significant positive association with AD-CM, even after controlling for potential confounders (Table 2 and Figure 1). Similarly, there was a significant AD-CM difference among serum UA strata. Post hoc comparison showed that high UA stratum showed significantly higher AD-CM than the other two strata (Table 3 and Figure 2A). Sensitivity analyses conducted only for CN subjects revealed very similar results (Tables 4, 5). Additional analyses to determine the mediating effects of age, sex, *APOE4*, VRS, or clinical diagnosis on the association between UA and AD-CM did not reveal any significant results (Table 6).

**TABLE 2:** Results of multiple logistic and linear regression analyses for assessing the relationship between serum UA and Aβ, AV-1451, AD-CM, or WMH volume in non-demented older adults.

	OR	95% CI	*P*
**Aβ positivity**			
Model 1	1.009	0.848 to 1.200	0.920
Model 2	1.010	0.838 to 1.217	0.916
Model 3	1.068	0.854 to 1.336	0.565

	**B**	**95% CI**	***P***

**Aβ retention, SUVR**			
Model 1	–0.002	−0.019 to 0.015	0.812
Model 2	0.001	−0.017 to 0.018	0.950
Model 3	0.001	−0.015 to 0.017	0.877
**AV-1451, SUVR**			
Model 1	–0.062	−0.189 to 0.065	0.334
Model 2	–0.070	−0.203 to 0.062	0.296
Model 3	–0.052	−0.168 to 0.064	0.377
**AD-CM, SUVR**			
Model 1	0.010	<0.001 to 0.020	0.043
Model 2	0.011	<0.001 to 0.021	0.042
Model 3	0.014	0.004 to 0.024	0.006
**WMH, cm^3^**			
Model 1	0.067	−0.354 to 0.489	0.754
Model 2	–0.031	−0.463 to 0.402	0.889
Model 3	–0.039	−0.488 to 0.410	0.865

*Abbreviations*:* UA, uric acid; Aβ, beta-amyloid; AD-CM, Alzheimer’s disease signature cerebral glucose metabolism; WMH, white matter hyperintensities; CI, confidence interval. The results of multivariate logistic or linear regression analyses are presented with OR B coefficient values, 95% CI and *P-*value. Global Aβ retention was used after natural log-transformation to achieve normal distribution. Model 1 did not include any covariates, model 2 included age and sex as covariates, and model 3 included all potential covariates, including age, sex, education, apolipoprotein ε4, vascular risk score, clinical diagnosis, serum albumin, body mass index, smoking status, and alcohol intake status.*

**TABLE 3:** Results of multiple logistic regression and general linear model analyses for assessing the relationship between serum UA strata and Aβ, AV-1451, AD-CM, or WMH volume in non-demented older adults.

	B (SE)	df	OR	95% CI	*p*
**Aβ positivity**					
Model 1	<0.001 (0.140)	1	1.000	0.760 to 1.315	1.000
Model 2	0.030 (0.152)	1	1.031	0.766 to 1.388	0.841
Model 3	0.145 (0.179)	1	1.156	0.814 to 1.643	0.417

	**Type III SS**	**df**	**MS**	**F**	***p***

**Aβ retention, SUVR**					
Model 1	0.009	2	0.005	0.086	0.918
Model 2	0.018	2	0.009	0.177	0.838
Model 3	0.075	2	0.037	0.969	0.380
**AV-1451, SUVR**					
Model 1	0.331	2	0.166	0.266	0.767
Model 2	0.442	2	0.221	0.350	0.706
Model 3	0.106	2	0.053	0.122	0.885
**AD-CM, SUVR**					
Model 1	0.123	2	0.062	3.602	0.028
Model 2	0.112	2	0.056	3.379	0.035
Model 3	0.144	2	0.072	4.668	0.010
**WMH, cm^3^**					
Model 1	48.975	2	24.396	0.833	0.436
Model 2	42.174	2	21.087	0.759	0.469
Model 3	41.082	2	20.541	0.725	0.485

*Abbreviations: UA, uric acid; Aβ, beta-amyloid; AD-CM, Alzheimer’s disease signature cerebral glucose metabolism; WMH, white matter hyperintensities; OR, odds ratio; CI, confidence interval; SS, square sum; MS, mean square. Global Aβ retention was used after natural log-transformation to achieve normal distribution. Model 1 did not include any covariates, model 2 included age and sex as covariates, and model 3 included all potential covariates, including age, sex, education, apolipoprotein ε4, vascular risk score, clinical diagnosis, serum albumin, body mass index, smoking status, and alcohol intake status.*

**TABLE 4:** Results of multiple logistic and linear regression analyses for assessing the relationship between serum UA and Aβ, AV-1451, AD-CM, or WMH volume in cognitive normal older adults.

	OR	95% CI	*p*
**Aβ positivity**			
Model 1	1.135	0.885 to 1.461	0.315
Model 2	1.077	0.826 to 1.403	0.584
Model 3	1.183	0.878 to 1.595	0.270

	**B**	**95% CI**	***p***

**Aβ retention, SUVR**			
Model 1	0.006	−0.018 to 0.021	0.398
Model 2	0.006	−0.009 to 0.021	0.454
Model 3	0.008	−0.008 to 0.023	0.324
**AV-1451, SUVR**			
Model 1	0.012	−0.032 to 0.057	0.587
Model 2	–0.003	−0.048 to 0.041	0.883
Model 3	–0.009	−0.060 to 0.042	0.721
**AD-CM, SUVR**			
Model 1	0.011	<0.001 to 0.022	0.042
Model 2	0.011	<0.001 to 0.022	0.044
Model 3	0.016	0.004 to 0.028	0.007
**WMH, cm^3^**			
Model 1	0.186	−0.313 to 0.684	0.465
Model 2	0.103	−0.415 to 0.622	0.695
Model 3	0.052	−0.496 to 0.600	0.853

*Abbreviations: UA, uric acid; Aβ, beta-amyloid; AD-CM, Alzheimer’s disease signature cerebral glucose metabolism; WMH, white matter hyperintensities. The results of multivariate logistic or linear regression analyses are presented with OR B coefficient values, 95% CI and *P*-value. Global Aβ retention was used after natural log-transformation to achieve normal distribution. Model 1 did not include any covariates, model 2 included age and sex as covariates, and model 3 included all potential covariates, including age, sex, education, apolipoprotein ε4, vascular risk score, serum albumin, body mass index, smoking status, and alcohol intake status.*

**TABLE 5:** Results of multiple logistic regression and general linear model analyses for assessing the relationship between serum UA strata and Aβ, AV-1451, AD-CM, or WMH volume in cognitive normal older adults.

	B (SE)	df	OR	95% CI	*p*
**Aβ positivity**					
Model 1	0.123 (0.211)	1	1.131	0.747 to 1.711	0.561
Model 2	0.071 (0.227)	1	1.073	0.687 to 1.675	0.756
Model 3	0.226 (0.250)	1	1.254	0.768 to 2.047	0.366

	**Type III SS**	**df**	**MS**	**F**	***p***

**Aβ retention, SUVR**					
Model 1	0.036	2	0.018	0.684	0.505
Model 2	0.044	2	0.022	0.886	0.414
Model 3	0.074	2	0.037	1.538	0.217
**AV-1451, SUVR**					
Model 1	0.083	2	0.042	0.700	0.500
Model 2	0.076	2	0.038	0.690	0.505
Model 3	0.129	2	0.065	1.126	0.331
**AD-CM, SUVR**					
Model 1	0.119	2	0.059	4.322	0.014
Model 2	0.115	2	0.057	4.223	0.016
Model 3	0.170	2	0.085	6.379	0.002
**WMH, cm^3^**					
Model 1	21.256	2	10.628	0.370	0.691
Model 2	16.261	2	8.130	0.289	0.749
Model 3	13.821	2	6.910	0.242	0.786

*Abbreviations: UA, uric acid; Aβ, beta-amyloid; AD-CM, Alzheimer’s disease signature cerebral glucose metabolism; WMH, white matter hyperintensities; OR, odds ratio; CI, confidence interval; SS, square sum; MS, mean square. Global Aβ retention was used after natural log-transformation to achieve normal distribution. Model 1 did not include any covariates, model 2 included age and sex as covariates, and model 3 included all potential covariates, including age, sex, education, apolipoprotein ε4, vascular risk score, serum albumin, body mass index, smoking status, and alcohol intake status.*

**TABLE 6:** Results of multiple linear regression analyses including the interaction term between serum UA strata and age (or gender or APOE4 or VR score) status predicting AD-CM for non-demented older subjects.

	B (95% CI)^a^	*P*
Serum UA	0.129(−0.004to0.261)	0.057
Age	0.001(−0.003to0.005)	0.563
Serum UA × Age	−0.002(−0.003to < 0.001)	0.107
Serum UA	< 0.001(−0.049to0.048)	0.988
Sex	−0.034(−0.105to0.037)	0.347
Serum UA × Sex	0.015(−0.017to0.046)	0.363
Serum UA	0.028(−0.017to0.074)	0.218
APOE4	−0.016(−0.091to0.058)	0.662
Serum UA × APOE4	−0.006(−0.040to0.028)	0.723
Serum UA	0.017(−0.006to0.041)	0.148
VRS	−0.024(−0.056to0.007)	0.131
Serum UA × VRS	0.003(−0.012to0.018)	0.700
Serum UA	0.049(0.005to0.093)	0.030
Clinical diagnosis	−0.019(−0.085to0.048)	0.581
Serum UA × Clinical diagnosis	−0.021(−0.052to0.010)	0.184

*Abbreviations: UA, uric acid; AD-CM, Alzheimer’s disease signature cerebral glucose metabolism; APOE4, apolipoprotein ε4; CI, confidence interval; VRS, vascular risk score. ^*a*^Multiple linear regression model included serum UA, age (or sex or APOE4 or VRS) and the interaction between serum UA and age (or sex or APOE4) treated as the independent variables; age, sex, education, APOE4, VRS, clinical diagnosis, were treated as covariates; and AD-CM treated as the dependent variable.*

### Association of Serum UA With Cognition

Word list recall *z*-scores were significantly different among the serum UA strata (Table 7). Post hoc comparison showed that the low UA stratum had a significantly lower WLR z-score than the other two strata (Figure 2B). MMSE z-scores showed marginally significant differences among UA strata (Table 7); post hoc comparison revealed that the MMSE z-score of the low UA stratum was significantly lower than that of the high stratum (Figure 2C).

**TABLE 7:** Results of general linear model analyses for assessing the relationship between serum UA strata and cognitive performance in non-demented older adults.

	Type III SS	df	MS	F	*P*
WLR z-Score	9.846	2	4.923	3.602	0.028
WLR z-Score^a^	4.675	2	2.338	1.822	0.163
MMSE z-Score	8.349	2	4.174	2.751	0.065
MMSE z-Score^a^	3.987	2	1.994	1.373	0.255

*Abbreviations: UA, uric acid; Aβ, beta-amyloid; AD-CM, Alzheimer’s disease signature cerebral glucose metabolism; WMH, white matter hyperintensities; SS, square sum; MS, mean square; WLR, Word List Recall; MMSE, Mini-Mental State Examination. Global Aβ retention was used after natural log-transformation to achieve normal distribution. ^*a*^To examine whether the association between UA and cognitive score is mediated by specific brain pathology, we included AD-CM which showed a significant relationship with serum UA as an additional covariate in the model for the association between UA vs. cognitive score.*

### Mediation by AD-CM for the Relationship Between Serum UA and Cognition

The relationship between UA strata and WLR (or MMSE) *z*-score was not statistically significant after AD-CM, which had a significant association with UA, was controlled as a covariate in the GLM analysis (Table 5).

## Discussion

In the present study of non-demented older adults, lower serum UA was associated with decreased AD-CM, but not with other AD neuroimaging biomarkers or WMH. There was also a significant positive association between serum UA and cognitive performance, which was mediated by AD-CM. To the best of our knowledge, this is the first study to reveal a relationship between serum UA and AD-CM, as well as between serum UA and cognitive performance.

We found a strong positive association of serum UA with AD-CM. Consistent with this result, previous animal cell culture and postmortem human brain cell studies showed that UA reduced Aβ-induced neuronal injury (Keller et al., 1998) and synaptic dysfunction in the AD brain (Ansari and Scheff, 2010), respectively. It is also well-known that UA has strong antioxidant characteristics (Ames et al., 1981; Miller et al., 1993; Choi et al., 2005; Gao et al., 2008). Preclinical studies indicated that UA may be protective against oxidative stress in the brain (Hooper et al., 1998; Bowman et al., 2010; Chen et al., 2012). In AD, oxidative stress is an early biological manifestation that plays an important role in its pathogenesis (Markesbery and Lovell, 2007; Moreira et al., 2008). Therefore, serum UA may act as a strong antioxidant to protect against AD-related synaptic dysfunction, which is closely related to brain hypometabolism (Terry et al., 1991; Mosconi et al., 2008). Although there have been few direct assessments of the effects of antioxidants on cerebral glucose metabolism in humans, many preclinical studies have shown that antioxidants have beneficial effects on brain glucose metabolism (Franzini et al., 2008; Bisbal et al., 2010; Picco et al., 2014).

Notably, we did not find any associations of serum UA with amyloid or tau pathologies. This indicates that the protective effect of UA against AD is not directly associated with the deposition of the two core AD proteins. Additionally, the presence of WMH, as a measure of cerebrovascular injury, was not associated with serum UA in the present study. This finding is not consistent with the results of a previous human study, which showed that elevated serum UA was associated with increased WMH volume (Schretlen et al., 2007). This discrepancy may be influenced by the fasting state before blood sampling for UA level measurement: in the present study, serum UA levels were measured in blood samples obtained after overnight fasting, whereas the prior study used non-fasting blood samples for UA level measurement. Non-fasting before blood tests may interfere with assessment of UA relationships because purine-containing diets can alter serum UA levels (Choi et al., 2005), leading to false positive results.

We examined the relationships of serum UA with MMSE, a measure of global cognition, and with WLR, a measure of episodic memory. Serum UA showed a significant positive association with WLR score and tended to show a positive association with MMSE score. Given that episodic memory decline is the earliest and most prominent change in AD (Howieson et al., 1997; Grober et al., 2000), the association between UA and WLR score indicates that lower UA may contribute to cognitive impairment beginning in the early stages of AD. When AD-CM was controlled as an additional covariate, the relationship between serum UA and WLR was no longer significant, indicating that decreased AD-CM may mediate the association between lower serum UA and episodic memory decline.

There were a few limitations in the present study. First, because this was a cross-sectional study, the association may not represent causality. For example, it is possible that the low serum UA may be the consequence of the cerebral glucose hypometabolism, or a sign of worse disease. It is known, for example, that weight loss often precedes the development of clinical dementia in AD patients (Gillette-Guyonnet et al., 2000; Johnson et al., 2006), and that a lower nutritional status could lead to a lower serum UA. It is interesting that a low BMI was associated with lower serum uric acid and with worse glucose hypometabolism in this study. Further long-term prospective studies are needed to clarify the etiological contribution of low UA to AD-related cerebral hypometabolism and cognitive decline. Second, the lack of repeated assessments of serum UA levels might have resulted in some errors in measurement of the serum levels because there are diurnal variations in serum UA levels (Andersen et al., 2015). However, such errors were minimized by ensuring that serum UA levels were assessed at the same time (8–9 a.m.) in all participants. Third, tau PET was applied after an average of 2.6 years from the baseline visit, whereas other neuroimaging scans were performed at the baseline. This temporal gap may have influenced the association between tau and UA. However, when we controlled for the temporal gap as an additional covariate, the results did not change. In addition, fewer participants underwent tau PET, relative to those who received other imaging modalities. This relatively reduced sample size may have decreased the statistical power and contributed to the negative result for tau deposition. Further studies with additional participants are needed to confirm these findings.

## Conclusion

The findings of the present study indicate that there is an association of low serum UA with AD-related cerebral hypometabolism, and whether this represents a causal relationship remains to be determined.

## Data Availability Statement

The data of the current study can be available from the independent data sharing committee of the KBASE research group on reasonable request. Requests for data access can be submitted to the administrative coordinator of the KBASE group by e-mail (kbasecohort@gmail.com).

## Ethics Statement

This study was approved by the Institutional Review Boards of Seoul National University Hospital (IRB No: C-1401-027547) and SNU-SMG Boramae Center (IRB No: 26-2015-60), Seoul, South Korea, and was conducted in accordance with the recommendations of the current version of the Declaration of Helsinki. All subjects or their legal representatives gave written and informed consent.

## Author Contributions

JK and DL conceived and designed the study. MB, DY, JL, SJ, KK, GJ, HL, J-YL, C-HS, Y-SL, SS, YK, and DL were involved in acquisition, or analysis and interpretation of the data and helped to draft the manuscript. JK, MB, DY, JL, and DL were major contributors in writing the manuscript and critically revising the manuscript for intellectual content and served as principal investigator and supervised the study. All authors read and approved the final manuscript.

## Conflict of Interest

The authors declare that the research was conducted in the absence of any commercial or financial relationships that could be construed as a potential conflict of interest.
